# Origins and developmental paths of medical conditions from mid-childhood to mid-adolescence in Australia: Early-life adverse conditions and their lasting effects

**DOI:** 10.1016/j.ssmph.2024.101717

**Published:** 2024-10-10

**Authors:** Lan Nguyen, Luke B. Connelly, Stephen Birch, Ha Trong Nguyen

**Affiliations:** a Centre for the Business and Economics of Health, The University of Queensland, Brisbane, Australia; b Department of Sociology and Business Law, The University of Bologna, Bologna, Italy; c Centre for Health Economics, University of Manchester, UK; d Centre for Health Economics and Policy Analysis, McMaster University, Canada; e The Kids Research Institute Australia, Perth, Australia; f Centre for Child Health Research, The University of Western Australia, Perth, Australia

## Abstract

This study investigates various common medical conditions affecting Australian children aged 4–14 years and the impact of prenatal and early-life conditions on these health conditions using a large national data set (n = 4122) with 15 years of follow-up. Consistent with the developmental origins of health and diseases hypothesis and the life-course models of health, the *in-utero* environment and parental financial hardship during pregnancy and shortly after birth play a significant role and have a lasting impact on the medical conditions of children. These significant effects are not reduced by controlling for child, family, and neighbourhood characteristics. The impact of improvements in family income when the child is aged 4–14 years does not compensate for the impact of health disadvantages in the prenatal and postnatal period.

## Highlights

• Analyses developmental trajectories of ongoing medical conditions in Australian children aged 4 to 14 years.
• Utilizes rigorous statistical models to investigate a representative population sample with 15 years of follow-up.
• Timing of financial stress in shaping child health: Early childhood poverty is particularly harmful to child health.
• Subsequent family income cannot mitigate health adversity in the early foundation years.

## 1 Introduction

In Australia, around 2 in 5 children aged 0–14 has one or more chronic conditions (Australian Institute of Health and Welfare–AIHW, 2023). These chronic illnesses significantly impact children's lives, interrupt a child's normal development, and tend to be long-lasting with persistent effects. The leading causes of the total burden of disease among children aged 0–14 are infant and congenital conditions, heart conditions, asthma, and mental health disorders (anxiety disorders, depressive disorders, conduct disorder, autism spectrum disorders) (AIHW, 2023).

Factors influencing child health are complex and multidimensional, including biological systems, individual psychological characteristics and health behaviours, socioeconomic determinants of health, and structural factors in a society that, together, interact and shape health outcomes (Spencer, 2018; Turney et al., 2013; Letourneau et al., 2013; Li et al., 2009; Irwin et al., 2007; Bradley & Corwyn, 2002; Lundberg, 1993).

According to the developmental origins of health and disease hypothesis, the sensitive periods of development, starting from in utero, can represent early major risk factors capable of influencing offspring development and have lifelong consequences, increasing disease risk later in life (Barker, 1995; Entringer et al., 2015; Eriksson, 2016; Gillman, 2005; Heindel & Vandenberg, 2015; Morton et al., 2016; O'Donnell & Meaney, 2017; Van den Bergh et al., 2020).

In addition to the developmental origins of health and diseases, the life course of health framework can offer substantial insight into the causes of health disparities by explaining how risk and protective factors at different stages of human development shape health throughout the life course (Braveman & Barclay, 2009; Halfon et al., 2014; Jones et al., 2019). Since health cannot be understood in terms of immediate environmental circumstances alone (Gowland, 2015), health trajectories are increasingly used to understand the developmental patterns and natural history of diseases, as a result of cumulative social and environmental exposures, both positive and negative (Halfon et al., 2014; Kuh & Shlomo, 2004; Halfon & Hochstein, 2002; Ben-Shlomo & Kuh, 2002).

Among the environmental factors that affect development, the family environment plays a powerful role in shaping the mental and physical health trajectories of children into adulthood as young children are dependent on their parents (van den Bergh et al., 2020; Turney et al., 2013). The early family economic conditions, especially early childhood poverty, may be particularly harmful to children's development (Duncan et al., 2012, 2017).

A large body of research has investigated the association between socioeconomic status (SES) and child health outcomes: much less studied is the timing of family financial stress in shaping child health and well-being (Clark et al., 2021) and few studies have examined trajectories of health status and its association with early life conditions (Haas, 2008). From an economic point of view, critical and sensitive periods in the development of the child relate to the costs and returns of remediation, and the later remediation is given to a disadvantaged child, the less effective it is (Cunha & Heckman, 2007; Currie, 2020; Heckman, 2007).

Applying the conceptual framework in the developmental origins of health and diseases and the life-course model of health, in this study, we assessed the impact of the *in-utero* environment and family financial hardship during pregnancy and shortly after birth on the developmental trajectories of medical conditions over mid-childhood to mid-adolescence in Australia, using a large-scale birth cohort survey. In addition, we investigated whether the SES status when the child aged 4–14 years modifies adverse health outcomes that seem to be already established earlier in response to an adverse *in-utero* environment and early exposure to family financial stress.

This paper makes three important contributions to the literature. First, to the best of our knowledge, this is the first study to assess the trajectories of ongoing medical conditions among Australian children from early childhood to mid-adolescence. We do so by estimating a class of group-based trajectory models to identify distinct clusters, or groups, of individuals who follow similar developmental trajectories of persistent medical conditions. We use different definitions of medical conditions as the sensitivity check. The presence of ongoing health problems is a more precise measure of health than a self-rated global health measure.

Second, this study adds more evidence of the critical period over a life course of health development to the existing literature. There has been no research into the association between pregnancy complications, early-life financial hardship, and the trajectories of ongoing medical conditions from childhood to young adulthood in the Australian context.

Lastly, we conduct robustness checks by comparing the results of trajectory models with those of traditional regression models (panel data analyses). This analysis utilizes an advanced econometric approach and a long panel dataset to address the concerns about endogeneity, given the potential two way-causality between family income and child health. The existing literature has explored the family income-child health gradient for young children in Australia and highlighted this unsolved issue due to data limitations (Khanam et al., 2014).

The rest of the paper is organized as follows: Section 2 describes methods and the dataset. Section 3 presents the results. Robustness checks appear in Section 4. Section 5 concludes.

## 2 Methods and data

### 2.1 Data

Data were drawn from the continuing Longitudinal Study of Australian Children (LSAC), a cohort survey and nationally representative for the Australian population from birth to adolescence. The first data were collected in 2004, and the follow-up surveys have been conducted every 2 years. At the baseline, the B cohort (birth cohort) and the K cohort (kindergarten cohort) were aged 0–1 years (5107 infants) and 4–5 years (4983 children), respectively.

The LSAC provides a very rich set of information on children's developmental outcomes and their family, community, and society characteristics. In this study, the LSAC's population weights were used to account for the complex survey design and to reflect the population level. Further information on the LSAC and the survey design is available from the LSAC's documentations and technical papers (Australian Institute of Family Studies-AIFS, 2023).

We used data from B cohort, wave 1 (2004) to wave 8 (2018), as this cohort provides information relating to the pregnancy period (wave 9 is the latest wave of the LSAC, however, we did not use data from this wave because of the possible disruptions caused by the Covid-19 pandemic). Birth risks and early financial conditions are two sets of factors that we hypothesize will predict future child health outcomes. Subgroups that reflect the medical condition trajectories since mid-childhood, once obtained, are hypothesized to be related to explanatory variables of *in-utero* and early childhood exposures (Herle et al., 2020).

The health outcomes are the parent-reported presence of ongoing medical conditions and the number of ongoing medical conditions for children aged 4–14 years. We did not utilize this information before 4 years old as traits of medical conditions are more apparent for parents to report at age 4 and above. In addition, maternal depression during pregnancy could be a significant factor in predicting child health outcomes and using child health information from 4 years old can overcome a possible reverse causality between a child's medical condition and maternal depression during pregnancy.

To improve the accuracy of the trajectory models, observations were only selected in the final sample if they provided at least 3 times of outcome measurement from wave 3 (aged 4 years) to wave 8 (aged 14 years) (Nguena Nguefack et al., 2020). Accordingly, the final samples used to analyse trajectories of the presence of ongoing medical conditions and the number of ongoing medical conditions are 4122 and 3930 individuals, respectively. In the robustness checks, we used 4481 individuals in the panel data regressions.

The attrition rates in the samples are a concern if non-response is systematically related to child health. We used the baseline data to compare participants who stayed in the main study (n = 4122) with those who left the study (n = 985) and no significant difference was found in the presence of medical conditions between the two groups. Further, when we changed the scope of attrition by analysing the different samples (4122 individuals, 3930 individuals, and 4481 individuals) in the analyses, our results remained comparable.

### 2.2 Models

First, we employed the group-based trajectory model (GBTM) to investigate and classify the developmental paths of child medical conditions over time. While traditional analytic methods for longitudinal datasets cannot identify the distribution, occurrence, and determinants of health and diseases in subpopulations with varying health trajectories, GBTM effectively addresses these challenges. This model can provide a life-course view of birth risks and early family economic conditions associated with poor health outcomes later in life.

GBTM uses maximum likelihood for the estimation of parameters and identifies distinct homogeneous clusters of individuals following a similar developmental trajectory on an outcome (Nagin & Odgers, 2010). The selection procedures to identify appropriate group trajectories were outlined by Jones and Nagin (2013). The GBMT model allows us to analyse the effects of time-stable covariates (risk factors) on the probability of group membership with the generalized logistic function and the effects of time-varying covariates on a trajectory where all parameter estimates are trajectory group specific.

We used Traj command (Jones & Nagin, 2013) in StataMP v.17 (StataCorp 2023) to conduct the analyses with different health outcomes. Traj allows the estimation of a wide range of distributions, including zero-inflated Poisson (e.g., where many individuals have no symptoms and the others experience one or more symptoms) and binary (e.g., classifications of whether or not an individual has met diagnostic criteria) outcomes (Nagin & Odgers, 2010). In our study, the trajectories of numbers of child medical conditions over the period 4–14 years were identified by GBTM with the zero-inflated Poisson (zip) model while the trajectories of the presence of child medical conditions (yes/no) were identified by GBTM with the logit model.

Next, for robustness checks, we used traditional analytical models including static panel data (random effects and fixed effects) and dynamic panel data (system GMM) to re-examine the effects of possible predictors of the presence of child medical conditions.

### 2.3 Health outcomes and explanatory factors

Child health outcomes are defined by (i) the presence of ongoing medical conditions and (ii) the number of ongoing medical conditions. The ongoing medical condition of a child was constructed in the survey by collecting parents’ responses (no/yes) to the question: “*Does the child have any of these ongoing conditions? (‘Ongoing conditions' exist for some period (weeks, months, or years) or re-occur regularly. They do not have to be diagnosed by a doctor.)”.*

The listed ongoing conditions include eczema, ADD (attention deficit disorder)/ADHD (attention deficit hyperactivity disorder), asthma, vision problems, diarrhoea/colitis, ear infections, constipation, soiling, hearing problems, tonsillitis, food or digestive allergies, frequent headaches, recurrent abdominal pain, recurrent pain in other parts of the body, bone, joint or muscle problem, anxiety disorder, depression, autism, asperges, or other autism spectrum, diabetes, epilepsy or seizure disorder, chronic fatigue, other infections, other illnesses. The numbers of “Yes” responses to the above medical conditions were coded as the count data in the LSAC.

All time-varying variables were collected from mid-childhood to mid-adolescence (children aged 4–14 years). Based on the literature, we used a standard set of explanatory variables to predict child health, including child characteristics, family factors, and neighbourhood environment. To identify time-constant factors associated with the likelihood of belonging to different trajectory subgroups which are established at baseline or before (Nagin & Odgers, 2010), we used the information at the study baseline (B0), including factors in the pregnancy period (birth weight, intensive care after birth, using instrument delivery, maternal asthma, and maternal depression) and family financial hardship in the period from pregnancy to child aged 1 year. These variables have been shown to be linked to child health outcomes in the literature (Rüdiger et al., 2019; Escobar et al., 2006; Keag et al., 2018; Liu et al., 2018; Propper et al., 2007; Kakinami et al., 2013). Child gender and Indigenous status are time-constant variables and were included in the set of risk factors.

There are other variables associated with child health outcomes in the literature, such as alcohol consumption and smoking in pregnancy, and breastfeeding. We included these variables in our preliminary regressions. The LSAC dataset lacks detailed information on the intensity and duration of these variables during pregnancy and after birth, and our preliminary results did not show a significant impact of these variables on child health outcomes, which contradicts established evidence in the literature. Therefore, we excluded these variables to avoid including too many insignificant factors in the models. We also excluded variables related to hypertension and small for gestational age from the models, as these are likely correlated with depression during pregnancy and intensive care after birth, respectively.

Financial distress was asked by the question “*Over the last* *12 months, due to shortage of money, have any of the following happened? Couldn't pay bills on time; Couldn't pay mortgage on time; Gone without meals; Been unable to heat or cool home; Pawned or sold something; Assistance from welfare/community org.; Limited childcare”.* The number of “yes” responses to these answers was used to construct a financial hardship scale in the LSAC survey: “0” denotes the least financial hardship and “6” denotes the most financial hardship. The information on financial distress may provide information over and above the income variable because we need to know both financial resources and the demands that are made upon them to understand whether individuals are in financial distress (Clark et al., 2021).

Family financial hardship from pregnancy to when the child was 1 year old was assessed separately as a baseline risk factor in the models. In addition, we collected family income at each time interval when the child was 4–14 years old. We assessed whether family income during this period significantly alters the developmental trajectories of long-term conditions or impacts child health disadvantages established during the prenatal and postnatal periods.

Along with financial distress during pregnancy and after birth, our variables of interest in the pregnancy are maternal asthma and depression. Data on maternal asthma and depression were collected from the questions relating to the pregnancy period “*What ‘over the counter’ medications were used? Asthma medications (Ventolin etc)? Yes/No*” and “*During this pregnancy, did you have problems with stress, anxiety, or depression? Yes/No*”. In Australia, asthma, hay fever, allergic rhinitis, anxiety-related problems, and psychological development problems are the four leading chronic conditions for children aged 0–14, while among all children aged 5–14, asthma is the leading cause of disease burden followed by mental health disorders (AIHW, 2022). These explanatory factors are hypothesized to be strongly positively correlated with child health outcomes in our study.

## 3 Results

### 3.1 Descriptive statistics

In our final sample of children aged 4 years, 51.22% were boys and 3.61% were Indigenous. In terms of birth risks, children with an instrumental delivery at birth, low birth weight, and receiving intensive care after birth were 38.40%, 5.60%, and 16.49%, respectively. The proportions of children of mothers who had asthma and depression during pregnancy were 5.23% and 15.11%, respectively. Mothers with an educational attainment of less than bachelor level comprised 39.61% of the sample. Families without both biological parents present constituted 13.52% of the family units in the sample. Table 1 presents the baseline summary of child, family, and neighbourhood characteristics.

**Table 1:** Baseline summary of child, family, and neighbourhood characteristics (n = 4122).

Variables	Proportion (%)	95% CI
**Child characteristics**		
Gender (male)	51.22	49.61–52.83
Indigenous status (yes)	3.61	3.03–4.30
Weight (not in normal weight range)	29.25	27.80–30.75
Physical activities (dislike)	7.25	6.44–8.16
Presence of ongoing medical conditions (yes)	34.15	32.62–35.72
Number of ongoing medical conditions (mean)	48.63	45.96–51.29
**Prenatal and postnatal risk factors**		
Birth weight (<2500 g)	5.60	4.89–6.41
Instrumental delivery at birth (yes)	38.40	36.85–39.98
Intensive care after birth (yes)	16.49	15.33–17.73
Maternal asthma during pregnancy (yes)	5.23	4.57–5.98
Maternal depression during pregnancy (yes)	15.11	14.00–16.29
Financial hardship score (mean)	50.10	46.93–53.26
**Family characteristics**		

Single biological parent in house	13.52	12.42–14.72
Log household size (mean)	1.47	1.46–1.48
Homeowner (no)	27.48	26.02–28.99
Maternal health (good/fair/poor)	7.68	6.84–8.60
Maternal depression score (mean)	9.17	9.05–9.29
Maternal education (<bachelor's degree)	39.61	38.03–41.21
**Family income**		
First quartile (highest income quartile)	26.91	25.52–28.33
Second quartile	24.34	23.00–25.74
Third quartile	24.17	22.81–25.57
Last quartile	24.58	23.19–26.04
**Neighbourhood characteristics (SEIFA)**		

First quartile (*most advantaged areas*)	25.92	24.52–27.37
Second quartile	25.00	23.62–26.43
Third quartile	24.54	23.16–25.97
Last quartile	24.54	23.15–25.98

(The baseline sample at B4 includes observations who were available at least 3 times in the LSAC survey from Wave 3 to Wave 8. SEIFA: Socio-Economic Indexes for Areas. All figures were weighted. 95% CI: 95% confidence interval.).

The lowest and highest number of medical conditions among children at the baseline were 0 and 8, respectively. Around 34.29% of children were reported to have the presence of at least one medical condition.

### 3.2 Presence of medical condition as health outcome

The selection of the GBTM model of medical condition was conducted in two stages. We identified the optimal number of groups in GBTM using the most used statistical criteria (the log-likelihood statistics) to evaluate model fit: Bayesian information criteria (BIC) and Akaike information criterion (AIC) (smaller values of BIC and AIC denote better models), the ability of the model to accurately separate the classes based on posterior probabilities (entropy >0.7, with higher values denoting better classification) (Nagin & Odgers, 2010) and the required sample size in the model (at least 7%) (Shahunja et al., 2022). We first assumed a degree of cubic polynomial type for age (Kim & Cho, 2022).

Next, after the optimal number of trajectory groups was determined, we reduced the degree of the polynomial function for each group (e.g., linear, quadratic) until the highest level of statistical significance was obtained for the polynomial coefficients (*p-value* <0.05). Based on the best 3 models with BIC, AIC, entropy criteria, and sample size, we further tested the adequacy of the selected models based on the average of the posterior probabilities (APP ≥ 0.7). All model fit statistics indicated that the model of three groups was the best fitting model for health outcomes as the presence of medical condition. Table 2 presents the model fit statistics.

**Table 2:** Summary of model fit statistics.

Number of groups	Polynomial distribution	BIC	AIC	Entropy	Lowest group in model (%)
Number of group selection with cubic polynomial distribution					
1	3	−15119.39	−15103.37		100
2	3 3	−13448.11	−13412.05	0.71	46.31
3	**3 3 3**	**−13285**	**−13228**	**0.65**	**22.37**
4	3 3 3 3	−13250	−13173.99	0.59	13.02
5	3 3 3 3 3	−13258.06	−13161.90	0.62	3.25(<7)

Three-group model with different polynomial distribution∗					Group 1 (APP, %)	Group 2 (APP,%)	Group 3 (APP,%)
3	**2 2 2**	**−13302.13**	**−13258.06**	**0.71**	**0.89**	**0.69**	**0.89**
3	1 2 3	−13303.66	−13259.59	0.71	0.91	0.66	0.90
3	2 2 3	−13303.07	−13254.99	0.71	0.91	0.66	0.90

(BIC: Bayesian information criterion; AIC: Akaike information criterion; APP: Average posterior probability. (∗): Table reports the three models with the lowest BIC, and AIC after conducting a range of polynomial distribution. Polynomial distribution: 3 = cubic, 2 = quadratic, 1 = linear).

**Fig. 1:**
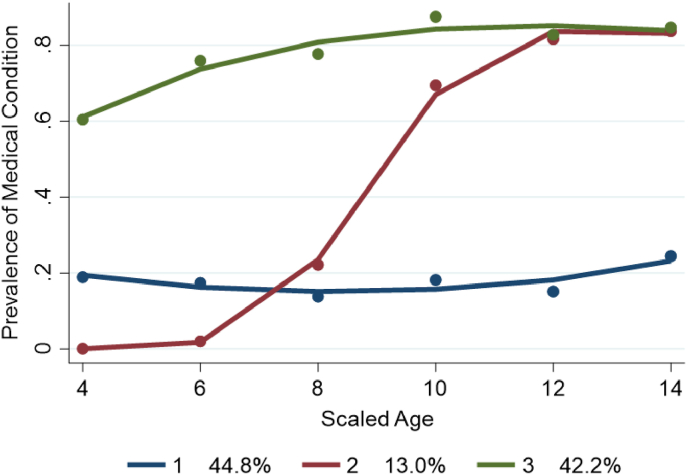
Presence of a medical condition.

Three distinct trajectories of medical conditions for children aged 4–14 years were found as follows (*p-value <*0.001): Group 1 with no medical condition (Persistently No, 44.8%); Group 2 has an emerging profile in medical conditions (Emerging, 13%) and Group 3 persistently has a medical condition(s) (Persistently Yes, 42.2%). Fig. 1 provides a graphical presentation of these three subgroups.

Fig. 1 shows the emergence of early and persistent gaps in the presence of medical conditions between Group 1 and Group 3. After identifying the distinct groups, we examined the impact of time-constant factors on the probability of belonging to a particular group and the impact of time-varying covariates on shifting these health trajectories. Adding time-varying covariates in the model allows the probability of trajectory group membership to vary as a function of individual-level characteristics (Nagin & Odgers, 2010). Table 3 presents the results.

**Table 3:** Risk factors predicting subgroup membership.

Risk factors (In pregnant and postnatal period)	Group 1 (Persistently No)	Group 2 (Emerging)	Group 3 (Persistently Yes)
		Coeff. (S.E)	Coeff. (S.E)
Gender	Reference Group	−0.103 (0.164)	0.006 (0.082)
Indigenous status		0.326 (0.441)	0.169 (0.257)
Instrumental delivery at birth		−0.025 (0.164)	0.179∗ (0.085)
Child intensive care after birth		−0.225 (0.248)	0.209 (0.118)
Birth weight (<2500 g)		−0.465 (0.443)	−0.232 (0.197)
Asthma in pregnancy		0.443 (0.379)	0.941∗∗ (0.191)
Depression in pregnancy		0.161 (0.249)	0.511∗∗ (0.117)
Financial hardship		0.081 (0.090)	0.133∗∗ (0.047)

(∗∗, ∗: Statistical significance level at 1% and 5%, respectively. All analyses were weighted. Standard errors in parentheses. Having a medical condition is coded as 1, 0 otherwise).

As presented in Table 3, maternal depression and asthma during pregnancy have long-term and enduring consequences on the child's medical conditions. Compared to Group 1, which has no persistent medical conditions, maternal asthma, and depression were significantly associated with the persistent presence of medical conditions in Group 3 (estimated coefficients are 0.511 (*p-value*<0.001) and 0.941 (*p-value*<0.001), respectively). This finding is in line with the fact that diseases of the respiratory system and mental/behavioral conditions are among the most common chronic illnesses in Australian children. Some studies have shown that poor control and increased severity of asthma during pregnancy are associated with childhood diseases, including those affecting the respiratory and nervous systems (Garcia-Sanchez et al., 2023; Liu et al., 2018; Martel et al., 2009). Effective management of asthma during pregnancy can have a significant impact on a child's future health (Clifton, 2019).

Additionally, there is a significant association (estimated coefficient is 0.133, *p-value*<0.001) between early-life financial hardship and the likelihood of belonging to Group 3. This suggests that health inequalities as the result of socioeconomic status begin as early as infancy. Previous studies in high-income countries have shown that family socioeconomic disadvantage in early childhood is associated with chronic diseases in later childhood. Spencer and Strazdins (2015) used LSAC data to demonstrate that children aged 6/7 from the lowest income quintile were more than twice as likely to develop their chronic disabling condition over the next four years compared to those from the highest income quintile. Using the Québec Longitudinal Study of Child Development, Kakinami et al. (2013) provided evidence that early and prolonged exposure to poverty significantly increases cardiovascular disease risk among 10-year-olds. Kakinami et al. (2014) demonstrated the impact of poverty on the likelihood of obesity and overweight among children in stable poor households compared to those in stable non-poor households. Additionally, Gauvin et al. (2007) found that children from families experiencing chronic poverty had more frequent asthma attacks and a higher cumulative health problems index score.

The correlation matrix reveals that the correlation between maternal asthma, maternal depression, and financial hardship is very low (the highest correlation coefficient is 0.12) in our dataset. When we rerun the GBTM model excluding each of these three variables, the results show only very marginal changes (relatively unchanged in the coefficient of maternal asthma) in these factors-related coefficients which are consistently positive and significant at the 1% level. The model with and without maternal depression shows the financial hardship coefficient changed from 0.133 to 0.154 (13.6%), suggesting that around 13.6% of the financial hardship effect on the presence of a child's medical condition seems to be mediated via maternal depression.

To explore the effects of time-varying factors on modifying the health trajectories, we added these factors into the model and presented the results in Table 4.

**Table 4:** Impact of time-varying factors on medical condition trajectories.

Time-varying covariates (Aged 4–14 years)	Group 1 (Persistently No)	Group 2 (Emerging)	Group 3 (Persistently Yes)
	Coeff. (S.E)	Coeff. (S.E)	Coeff. (S.E)
Child weight (not in normal weight range)	0.132 (0.079)	0.085 (0.268)	0.112 (0.081)
Child physical activities (dislike)	0.245∗ (0.111)	0.500 (0.332)	0.312∗∗ (0.117)
Family structure (bio-parents not co-habit)	−0.011 (0.123)	0.146 (0.285)	0.046 (0.117)
Log Household size	−0.555∗∗ (0.165)	−0.542 (0.448)	−0.338∗ (0.172)
Homeowner (No)	0.104 (0.094)	−0.127 (0.264)	0.028 (0.097)
Mother's education (<bachelor's degree)	−0.019 (0.081)	0.483∗ (0.219)	0.029 (0.084)
Mother's physical health (good/fair/poor)	0.298∗ (0.132)	0.211 (0.431)	0.163 (0.122)
Mother's depression	0.044∗∗ (0.011)	0.125∗∗ (0.047)	0.035∗∗ (0.012)
Quartile family income	−0.017 (0.037)	−0.155 (0.103)	−0.056 (0.037)
Quartile SEIFA	−0.019 (0.037)	0.090 (0.097)	0.070∗ (0.036)

(∗∗,∗: Statistical significance level at 1% and 5%, respectively. All analyses were weighted. Standard errors in parentheses. Having a medical condition is coded as 1, 0 otherwise).

As shown by Table 4, the health trajectories of children aged 4–14 years were found to be uncorrelated with contemporaneous family income. In other words, the contemporaneous family income neither eliminates nor attenuates the health gaps of the subgroups that were established in the earlier period and has no significant association with the emerging profile of medical conditions (Group 2). These findings were unchanged when we used alternative measures of income (continuous income & log income).

Among the time-varying factors, a mother's depression appears to have a significant impact (*p-value*<0.001) in varying health trajectories in all subgroups. The largest size effect of maternal depression was found in Group 2 consisting of children developing medical conditions gradually. A reserve causality may exist in the link between the mother's depression and the child's health outcome, however, Group 1, which consists of children without persistent medical conditions shows that having a mother with depression was associated with the likelihood of shifting the health trajectories upward. The negative impact of maternal mental health on child health and development has been demonstrated in the literature (Coles & Cage, 2022; Kingston & Tough, 2014; Le & Nguyen, 2017, 2018; Propper et al., 2007).

### 3.3 Number of medical conditions as health outcome

To examine whether birth risk factors and early-life economic conditions are important determinants of co-morbidities, we repeated the analyses using the number of ongoing medical conditions as an alternative health outcome.

The required steps to select the model fit were repeated as in the main study. All model statistics indicate the best fitting model has three groups (negative BIC, AIC closest to zero, entropy>0.70; APP≥0.7, none of the group membership below 7%). Table 5 presents the model fit statistics.

**Table 5:** Summary of model fit statistics.

Number of groups	Polynomial distribution	BIC	AIC	Entropy	Lowest group in model (%)
Number of group selection with cubic polynomial distribution					
1	3	−27331.77	−27319.22	.	100
2	3 3	−24005.73	−23977.49	0.78	31.27
**3**	**3 3 3**	**−23443.24**	**−23399.31**	**0.74**	**8.21**
4	3 3 3 3	−23365.30	−23305.67	0.66	3.81 (<7)

Three-group model with different polynomial distribution∗					Group 1 (APP, %)	Group 2 (APP,%)	Group 3 (APP,%)
**3**	**2 2 2**	**−23437.59**	**−23403.07**	**0.74**	**0.87**	**0.88**	**0.87**
3	1 3 2	−23438.28	−23403.76	0.74	0.87	0.89	0.88
3	3 3 2	−23439.11	−23398.31	0.74	0.87	0.88	0.88

(BIC: Bayesian information criterion; AIC: Akaike information criterion; APP: Average posterior probability. (∗): Table reports the three models with the lowest BIC, and AIC after conducting a range of polynomial distribution. Polynomial distribution: 3 = cubic, 2 = quadratic, 1 = linear).

**Fig. 2:**
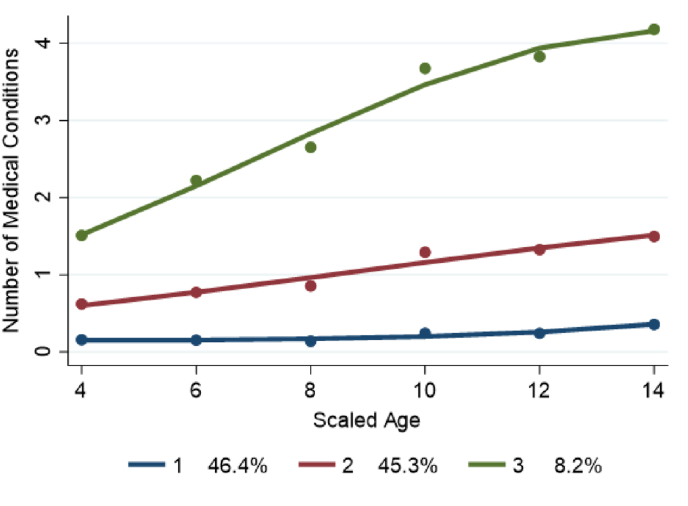
Trajectories of subgroups based on the number of ongoing medical conditions.

The developmental course of child health over time in terms of the number of medical conditions is visualized in Fig. 2. The subgroups were found to be as follows (*p-value<*0.001): 46.4% were classified as having no medical conditions reported (Group 1), 45.3% of children having one medical condition reported (Group 2), and 8.2% of children belonged to a group with more than 2 medical conditions reported (Group 3).

Fig. 2 shows the early and continuing divergent patterns of a child's ongoing health conditions. Table 6, Table 7 present evidence of the persistent effects of the utero environment and early financial hardship on the subgroups.

**Table 6:** Risk factors predicting subgroup membership.

Risk factors (In pregnancy and postnatal period)	Group 1 (No medical conditions)	Group 2 (1 medical condition)	Group 3 (>1 medical condition)
		Coeff. (S.E)	Coeff. (S.E)
Gender (Male)	Reference Group	−0.001 (0.089)	−0.109 (0.146)
Indigenous status (Yes)		0.385 (0.289)	0.420 (0.370)
Instrumental delivery at birth		0.115 (0.091)	0.284 (0.149)
Child intensive care after birth		0.161 (0.130)	0.391 (0.196)∗
Birth weight (<2500 g)		−0.283 (0.217)	−0.293 (0.337)
Asthma in pregnancy		0.761∗∗ (0.208)	1.398∗∗ (0.269)
Depression in pregnancy		0.395∗∗ (0.134)	1.033∗∗ (0.174)
Financial hardship		0.135∗ (0.055)	0.289∗∗ (0.066)

(∗∗,∗: Statistical significance level at 1% and 5%, respectively. All analyses were weighted.

Standard errors in parentheses).

**Table 7:** Impact of time-varying factors on the number of medical condition trajectories.

Time-varying covariates (Aged 4–14 years)	Group 1 (No medical conditions)	Group 2 (1 medical condition)	Group 3 (>1 medical condition)
	Coeff. (S.E)	Coeff. (S.E)	Coeff. (S.E)
Child weight (not in normal weight range)	0.184∗ (0.072)	0.114∗∗ (0.030)	0.106∗ (0.052)
Child physical activities (dislike)	0.314∗∗ (0.096)	0.097∗ (0.038)	−0.044 (0.047)
Family structure (bio-parents do not cohabit)	−0.029 (0.117)	0.005 (0.075)	0.032 (0.102)
Log Household size	−0.481∗∗ (0.158)	−0.150∗ (0.072)	−0.182∗ (0.089)
Homeowner (No)	0.007 (0.091)	0.009 (0.056)	0.076 (0.098)
Mother's education (<bachelor's degree)	−0.022 (0.076)	0.036 (0.036)	0.050 (0.053)
Mother's physical health (good/fair/poor)	0.247∗ (0.105)	0.061 (0.064)	0.082 (0.055)
Mother's depression	0.034∗∗ (0.009)	0.029∗∗ (0.005)	0.038∗∗ (0.009)
Quartile family income	0.021 (0.034)	0.013 (0.014)	−0.000 (0.018)
Quartile SEIFA	−0.012 (0.036)	0.034 (0.017)	−0.021 (0.021)

(∗∗,∗: Statistical significance level at 1% and 5%, respectively. All analyses were weighted.

Standard errors in parentheses. Having a medical condition is coded as 1, 0 otherwise).

Table 6 provides strong evidence of an early adverse environment in predicting child medical conditions, which drives the morbidity distribution unevenly among children. Having stress and asthma during pregnancy are significant determinants (*p-value*<0.001) of being a member of Groups 2 and 3. Further, there is an approximately two-fold increase in the effect sizes of these factors in Group 3 compared to that in Group 2.

Additionally, the results suggest that early-life financial hardship was a significant predictor of belonging to Group 2 (coefficient is 0.135, *p-value*<0.05) or Group 3 (coefficient is 0.289, *p-value*<0.001), relative to Group 1. Early financial distress was associated with less optimal growth during infancy and children's formative and crucial early years.

Our results in Table 7 imply that differences in the contemporaneous family income do not explain the trajectories of ongoing medical conditions for children in our sample. This finding again confirms that once medical conditions are established from early childhood, it appears to be lasting even if SES status changes favourably in later periods. Using different income-based measures (continuous & log income) does not alter this finding.

By contrast, among other time-varying factors, these divergent patterns are significantly associated with maternal mental health (*p-value*<0.001), increasing the likelihood of upward shifting the medical condition trajectories. Negative effects of the mental health of mothers on child health have been documented in previous studies, due to low parenting capability, less attention to their children leading to more accidents and injuries, failure to identify illness symptoms in their children early to seek medical advice in time or being inconsistent in their use of preventative care measures (Propper et al., 2007; Yeung et al., 2002).

## 4 Robustness checks

So far, we have reported the trajectories of medical conditions over time using maximum likelihood estimates by GBTM. Although GBTM has been increasingly applied for its usefulness in identifying homogeneous subpopulations that follow distinct health trajectories over time, a limitation of GBTM is that it assumes no variation between individuals in the same class (Nagin & Odgers, 2010; Nguena Nguefack et al., 2020).

We conducted the robustness checks of our findings by the traditional analytic approaches, allowing the variation between units. We also collected more observations in these regression methods as individuals are not required to appear in at least three waves of the LSAC survey. The estimated models are as follows:

(1) H_it_ = α + βX_it_ + π_i_ + ε_it_

(2) H_it_ = α’ + δH_it-1_ + β′X_it_ + π_i_ + μ_it_

Where: H_it_ is the presence of the child's medical condition, X_it_ is a set of the same time-varying covariates (child aged 4–14 years) used in our previous analysis, π_i_ presents child observable time-constant factors, ε_it,_ and μ_it_ are error terms, i denotes individual child and t represents year from 2008 to 2018. We treated poor utero environment factors and early financial hardship as time-constant explanatory variables in our models. The family background characteristics and adverse childhood conditions measured at baseline were treated as time-constant covariates in prior studies (Lee & Jackson, 2017; Smith & Smith, 2010; South & Crowder, 1999).

Generalized linear models (pooled cross-sectional data, random effects, and fixed-effects panel data) were used to estimate equation (1). The random effects model can account for time-constant covariates while the fixed-effects model can control for all unobserved and observed time-constant confounders.

To address our concern regarding the possible endogeneity of parental income in Equation (1) and the fact that health has a stock component, we extend our models to the dynamic panel data in Equation (2) where we treat income as an endogenous variable using two-step system generalized method of moments (GMM) (Arellano & Bond, 1991; Blundell & Bond, 1998).

Our results across the different specifications are robust and show the long-run impacts of *in-utero* and adverse early-life financial hardship on the ongoing health conditions of children and young people. Table 8 presents the regression results.

**Table 8:** Regression results (family income was treated as an exogenous variable).

Variables	Pooled data analysis Coeff. (Robust S.E)	Random effects panel data analysis Coeff. (Robust S.E)	Fixed-effects panel data analysis Coeff. (Robust S.E)
**Child characteristics**			
Gender (male)	−0.003 (0.007)	0.000 (0.010)	–
Indigenous status (Yes)	0.020 (0.021)	−0.010 (0.028)	–
Child weight (not in normal weight range)	0.027∗∗ (0.008)	0.018∗ (0.008)	0.012 (0.010)
Child physical activities (dislike)	0.079∗∗ (0.011)	0.048∗∗ (0.010)	0.029∗ (0.012)
Age (in years)	0.024∗∗ (0.001)	0.024∗∗ (0.001)	0.024∗∗ (0.001)
**Family characteristics**			

Family structure (bio-parents do not cohabit)	0.013 (0.010)	0.018 (0.013)	0.029 (0.020)
Log Household size	−0.077∗∗ (0.014)	−0.040∗ (0.018)	0.005 (0.030)
Homeowner (No)	−0.004 (0.009)	0.003 (0.010)	−0.007 (0.014)
Mother's education (<bachelor's degree)	0.004 (0.007)	0.010 (0.009)	0.011 (0.018)
Mother's physical health (good/fair/poor)	0.061∗∗ (0.013)	0.036∗∗ (0.012)	0.007 (0.015)
Mother's depression	0.010∗∗ (0.001)	0.007∗∗ (0.001)	0.003∗ (0.001)
**Quartile family income***(Ref: Highest quartile income)*			
Second quartile	0.017 (0.010)	0.005 (0.010)	−0.007 (0.012)
Third quartile	0.033∗∗ (0.010)	0.014 (0.011)	−0.009 (0.013)
Last quartile	−0.004 (0.011)	−0.010 (0.011)	−0.026 (0.014)
**Neighbourhood characteristics**			

Quartile SEIFA *(Ref: Most advantaged area)*			
Second quartile	−0.012 (0.010)	−0.013 (0.011)	−0.016 (0.015)
Third quartile	−0.007 (0.010)	−0.002 (0.012)	−0.001 (0.017)
Last quartile	0.000 (0.010)	0.003 (0.012)	0.015 (0.020)
**Risk factors (in pregnant and postnatal period)**			

Instrumental delivery at birth	0.018∗ (0.007)	0.018 (0.011)	–
Child intensive care after birth	0.026∗ (0.010)	0.035∗ (0.015)	–
Birth weight (<2500 g)	−0.039∗ (0.017)	−0.039 (0.025)	–
Asthma in pregnancy	0.139∗∗ (0.015)	0.120∗∗ (0.023)	–
Depression in pregnancy	0.057∗∗ (0.010)	0.067∗∗ (0.014)	–
Financial hardship	0.010∗ (0.004)	0.013∗ (0.006)	–
Observation	21,943	21,943	21,943

(∗∗, ∗: Statistical significance level at 1% and 5%, respectively. All analyses were clustered at the individual level. Pooled and fixed-effects analyses were weighted. Robust standard errors are in parentheses.

Having a medical condition is coded as 1, 0 otherwise).

Table 8 shows the consistent findings obtained by panel data analyses compared to that by GBTM and emphasizes the powerful roles of the early environment in predicting the presence of child medical conditions. The likelihood of having medical conditions is significantly increased for those children whose mothers experienced asthma or depression during pregnancy. Additionally, early financial hardship was a significant determinant of child health conditions while the concurrent family income was found to be an insignificant factor. This finding corroborates those of another study conducted on children in the UK (Clark et al., 2021).

We measured the degree of multicollinearity of all independent variables by the variance inflation (VIF) which all were less than 2.0, much lower than the ‘rule of thumb’ VIF of 5 or 10 (O’brien, 2007), suggesting that the multicollinearity problem is not a concern in our estimates.

Table 9 presents the results obtained by the two-step system GMM where family income was treated as an endogenous variable. Based on the second-order autocorrelation test (AR (2)) and the Hansen test on overidentifying restrictions, we use two lags of medical condition to fit the model.

**Table 9:** Dynamic panel-data estimation by two-step system GMM (Family income was treated as an endogenous variable).

Variable Child characteristics	Dynamic panel-data analysis Coeff. (Corrected S.E)		
	Model 1 (Without risk factors)	Model 2 (Without financial hardship)	Model 3 (All variables)
Medical condition_t-1_	0.258∗∗ (0.016)	0.257∗∗ (0.016)	0.256∗∗ (0.016)
Medical condition_t-2_	0.061∗∗ (0.015)	0.061∗∗ (0.015)	0.060∗∗ (0.015)
Gender (male)	−0.006 (0.009)	−0.007 (0.009)	−0.007 (0.009)
Indigenous status (yes)	0.040 (0.029)	0.044 (0.028)	0.039 (0.028)
Child weight (not in normal weight range)	0.019∗ (0.009)	0.019∗ (0.009)	0.018∗ (0.009)
Child physical activities (dislike)	0.070∗∗ (0.012)	0.069∗∗ (0.012)	0.069∗∗ (0.012)
Aged 10	0.120∗∗ (0.010)	0.121∗∗ (0.010)	0.121∗∗ (0.010)
Aged 12	0.067∗∗ (0.011)	0.068∗∗ (0.011)	0.068∗∗ (0.011)
Aged 14	0.107∗∗ (0.011)	0.108∗∗ (0.011)	0.108∗∗ (0.011)
**Family characteristics**			

Family structure (bio-parents do not cohabit)	0.022 (0.013)	0.018 (0.013)	0.016 (0.013)
Log Household size	−0.044∗ (0.018)	−0.041∗ (0.018)	−0.044∗ (0.018)
Homeowner (No)	0.011 (0.012)	0.011 (0.012)	0.008 (0.012)
Mother's education (<bachelor's degree)	0.006 (0.009)	0.005 (0.009)	0.004 (0.009)
Mother physical health (good/fair/poor)	0.037∗∗ (0.014)	0.034∗ (0.014)	0.033∗ (0.014)
Mother's depression	0.008∗∗ (0.001)	0.007∗∗ (0.001)	0.007∗∗ (0.001)
**Quartile family income** (*Ref: Highest quartile income)*			
Second quartile	0.007 (0.019)	0.004 (0.019)	0.005 (0.019)
Third quartile	−0.011 (0.021)	−0.014 (0.021)	−0.014 (0.021)
Last quartile	−0.039 (0.022)	−0.041 (0.022)	−0.041 (0.022)
**Neighbourhood characteristics**			

Quartile SEIFA *(Ref: Most advantaged area*)			
Second quartile	−0.013 (0.013)	−0.012 (0.013)	−0.012 (0.013)
Third quartile	−0.006 (0.013)	−0.004 (0.013)	−0.006 (0.013)
Last quartile	0.004 (0.014)	0.008 (0.014)	0.005 (0.014)
**Risk factors (in pregnant and postnatal period)**			

Instrumental delivery at birth	–	0.009 (0.010)	0.010 (0.010)
Child intensive care after birth	–	0.023 (0.013)	0.023 (0.013)
Birth weight (<2500 g)	–	−0.023 (0.023)	−0.024 (0.023)
Asthma in pregnancy	–	0.087∗∗ (0.019)	0.085∗∗ (0.019)
Depression in pregnancy	–	0.047∗∗ (0.013)	0.044∗∗ (0.013)
Financial hardship	–	–	0.013∗ (0.005)

AR (1) (p-value)	0.000	0.000	0.000
AR (2) (p-value)	0.566	0.585	0.583
Hansen test (p-value)	0.260	0.303	0.312

Observation	13,276	13,276	13,270

(∗∗, ∗: Statistical significance level at 1% and 5%, respectively. Corrected standard errors in parentheses. The number of instruments in Model 1, Model 2, and Model 3 are 68, 73, and 74, respectively. Having a medical condition is coded as 1, 0 otherwise).

Table 9 shows that none of the coefficients on the contemporaneous family income is statistically significant in all models that control for a large set of time-varying covariates and early-life background measures. By contrast, the significant impact of *in-utero* and early-life financial hardship on later-life health is apparently shown in the results. Adding financial hardship in the model (3) only changes the estimated coefficients of maternal asthma and depression in pregnancy marginally.

## 5 Conclusion

This study assessed the early risk factors and the trajectories of ongoing medical conditions of Australian children over a 14-year follow-up within a population-based birth cohort, using a group-based trajectory model. We find substantial and lasting effects of an adverse *in-utero* environment as represented by maternal asthma and maternal depression, and economic hardship condition during pregnancy and infancy on subsequent medical conditions of children and young adolescents. Our findings hold true when all time-varying factors of child, family, and neighbourhood are included simultaneously in the static and dynamic panel data models. While the early adverse conditions are found to be significantly associated with child illness later in life, contemporaneous family income has no significant impact on these health conditions.

There are several limitations of the work reported in this paper. First, our study used the medical condition information that is parent-reported, and these medical conditions do not have to be diagnosed by a doctor. Second, we used cohort data, and the results might be affected by a cohort or period effect. Despite these limitations, this study provides robust evidence about the impact of early adversity on following child health by using a nationally representative survey. In addition, the reported health conditions exist for some period (weeks, months, or years) or re-occur regularly: this may serve to mitigate the self-reporting bias in health status observed in specific time points and be more precise than self-rated global health measure. Our findings are consistent with different methods, observations, and health outcome definitions.

Overall, our study demonstrates the consequences of early adversity during gestation and infancy on the presence of ongoing medical conditions for children and young adolescents. Many chronic diseases can be traced back to childhood deprivation (Newhouse, 2021). The findings suggest future research might seek to explore the impact of early childhood interventions on child health. Early interventions have significant potential to improve the health of children and young adolescents.

## CRediT authorship contribution statement

**Lan Nguyen:** Writing – review & editing, Writing – original draft, Methodology, Formal analysis, Data curation, Conceptualization. **Luke B. Connelly:** Writing – review & editing, Validation, Supervision, Methodology, Funding acquisition, Conceptualization. **Stephen Birch:** Writing – review & editing, Validation, Supervision, Methodology, Funding acquisition, Conceptualization. **Ha Trong Nguyen:** Writing – review & editing, Validation, Supervision, Methodology, Funding acquisition, Conceptualization.

## Ethical issues

The ethical approval for the LSAC data was from the Australian Institute of Family Studies Ethics Committee. There was no further need to get ethical approval as this study used an anonymised and unrestricted secondary dataset.

## Funding

This work was supported by the Australian Research Council Discovery Project [Project ID: DP200103049].

This study uses data from Growing Up in Australia, the Longitudinal Study of Australian Children (LSAC). The LSAC is conducted in partnership between the Department of Families, Housing, Community Services, and Indigenous Affairs (FaHCSIA), the Australian Institute of Family Studies (AIFS) and the Australian Bureau of Statistics (ABS). The findings and views reported in this paper are those of the authors and should not be attributed to FaHCSIA, AIFS or the ABS.

## Declaration of competing interest

The authors declare no conflicts of interest.

## Data Availability

The authors do not have permission to share data.
